# Clinical features of patients with MOG-IgG associated disorders and analysis of the relationship between fibrinogen-to-albumin ratio and the severity at disease onset

**DOI:** 10.3389/fneur.2023.1140917

**Published:** 2023-04-20

**Authors:** Yue Li, Sai Wang, Panpan Liu, Jinxiu Ma, Xinjing Liu, Jing Yuan

**Affiliations:** Department of Neurology, The First Affiliated Hospital of Zhengzhou University, Zhengzhou, China

**Keywords:** MOG-IgG associated disorders, clinical features, magnetic resonance imaging, fibrinogen, albumin, expanded disability status scale

## Abstract

**Objective:**

The study aimed to investigate the differences in clinical features between pediatric and adult patients with first-episode MOG-IgG associated disorders (MOGAD) and evaluate the relationship between the fibrinogen-to-albumin ratio (FAR) and the severity of neurological deficits at disease onset.

**Methods:**

We retrospectively collected and analyzed biochemical test results, imaging characteristics, clinical manifestations, expanded disability status scale (EDSS) score, and FAR. The Spearman correlation analysis and logistic regression models were used to examine the association between FAR and severity. Receiver operating characteristic (ROC) curve analysis was to analyze the predictive ability of FAR for the severity of neurological deficits.

**Results:**

Fever (50.0%), headache (36.1%), and blurred vision (27.8%) were the most common clinical manifestations in the pediatric group (<18 years old). However, in the adult group (≥18 years old), the most common symptoms were blurred vision (45.7%), paralysis (37.0%), and paresthesia (32.6%). Fever was more common in the pediatric group, while paresthesia was more common in the adult patients, with all differences statistically significant (*P* < 0.05). The most frequent clinical phenotype in the pediatric group was acute disseminated encephalomyelitis (ADEM; 41.7%), whereas optic neuritis (ON; 32.6%) and transverse myelitis (TM; 26.1%) were more common in the adult group. The differences in clinical phenotype between the two groups were statistically significant (*P* < 0.05). In both pediatric and adult patients, cortical/subcortical and brainstem lesions were the most common lesions on cranial magnetic resonance imaging (MRI), whereas, for spinal MRI, cervical and thoracic spinal cord lesions were the most commonly observed. According to binary logistic regression analysis, FAR was an independent risk factor for the severity of neurological deficits (odds ratio = 1.717; 95% confidence interval = 1.191–2.477; *P* = 0.004). FAR (*r* = 0.359, *P* = 0.001) was positively correlated with the initial EDSS score. The area under the ROC curve was 0.749.

**Conclusion:**

The current study found age-dependent phenotypes in MOGAD patients as ADEM was more commonly observed in patients < 18 years old, while ON and TM were more frequently found in patients ≥18 years old. A high FAR level was an independent indicator for more severe neurological deficits at disease onset in patients with a first episode of MOGAD.

## 1. Introduction

Myelin oligodendrocyte glycoprotein (MOG) is produced by oligodendrocytes, which are the myelin-forming cells of the central nervous system (CNS). MOG is preferentially expressed on the outermost layers of the myelin sheath and surface of the oligodendrocytes, accounting for about 0.05% of the total myelin proteins, and it is also a vital surface marker of oligodendrocyte maturation (1, 2). Its specific location on the outermost layer of the myelin sheath makes it a potential target for autoimmune antibodies and cell-mediated responses during demyelination (2, 3). The MOG antibody is a subtype of immunoglobulin (Ig)G1 that can effectively regulate complement-dependent cytotoxicity. MOG-IgG associated disorders (MOGAD), which is an inflammatory demyelinating disease of the CNS, is mediated by anti-MOG antibodies. MOGAD phenotypes vary and include optic neuritis (ON), transverse myelitis (TM), acute disseminated encephalomyelitis (ADEM), and meningeal/brainstem encephalitis. Although the clinical presentation of this disease sometimes resembles neuromyelitis optica spectrum disorders (NMOSD), MOGAD is definitely considered a distinct disease with immunopathological features markedly different from those of multiple sclerosis (MS) and NMOSD (4, 5). A growing body of research suggests that MOGAD is an individual spectrum of disease (4–7). In 2018, MOGAD was internationally recommended as an independent disease (8, 9). Recently, international MOGAD panel have proposed diagnostic criteria for MOGAD (10).

Fibrinogen (FIB) is a 340-kDa soluble glycoprotein synthesized by hepatocytes (11, 12). It is not only an essential component of the coagulation cascade response but also an acute-phase reactant that reflects the systemic inflammatory state (13, 14). Albumin (ALB), also synthesized in the liver, is the most abundant protein in the plasma and has significant anti-inflammatory activity (15, 16). FIB and ALB are widely used as essential markers in the response against systemic inflammation and hemorheological changes, while the FIB-to-ALB ratio (FAR) includes these two indicators, which is a valuable serological index. As reported, FAR may serve as a valuable systemic inflammatory and disease activity marker in autoimmune diseases, such as rheumatoid arthritis (17). Therefore, based on the combined analysis of FIB and ALB, we hypothesized that FAR could be used as a predictor for the severity of neurological deficits at disease onset in patients with the first episode of MOGAD.

This study analyzed the clinical, laboratory, and magnetic resonance imaging (MRI) characteristics of patients with first-attack MOGAD. This study investigated the relationship between FAR and severity at disease onset and the predictive value of FAR to identify and judge the severity of the disease at an early stage. Our results provide a basis for subsequent standardized treatment and management.

## 2. Materials and methods

### 2.1. Study cohort

Data from 82 MOGAD patients admitted to the First Affiliated Hospital of Zhengzhou University between January 2018 and June 2022 were analyzed in this retrospective study.

All MOGAD patients included in this study met the international recommendations of Jarius et al. (8) for the diagnosis of MOG encephalomyelitis: (1) positive serum MOG-IgG test based on live cell-based assays (CBA) using full-length human MOG as a target antigen; (2) presence of one or a combination of the following clinical manifestations: ON, TM, encephalitis or meningoencephalitis, and brainstem encephalitis; (3) MRI or electrophysiological (visual-evoked potentials in patients with isolated ON) findings related to CNS demyelination; (4) exclusion of alternative diagnoses. The following exclusion criteria were applied: non-first episode MOGAD, patients with previous diseases that severely affected motor ability and visual function (e.g., trauma and acute cerebrovascular disease), use of corticosteroids or immunosuppressive therapies within 3 months prior to admission, complicated serious diseases (e.g., liver disease, renal disease, cardiovascular disease, and malignant tumor), and patients who were double positive for anti-MOG-IgG and anti-aquaporin-4 (AQP-4)-IgG antibodies.

This study was approved by the Ethics Committee of the First Affiliated Hospital of Zhengzhou University. We strictly complied with the Declaration of Helsinki and anonymized all patient data.

### 2.2. Clinical data collection

Clinical information, laboratory results, and radiological records were collected and documented, including age at onset, sex, clinical presentations, laboratory tests (routine blood tests, coagulation tests, liver function, renal function, blood lipids, and cerebrospinal fluid analysis), MRI findings at admission, and expanded disability status scale (EDSS) scores at admission and discharge. FAR was calculated using the following formula: *FIB* × 100%/*ALB*.

Patients were categorized into pediatric (<18 years old) and adult (≥18 years old) groups based on age at disease onset. Differences in clinical, laboratory, and imaging characteristics between the two groups were analyzed. Patients were then divided into mild-to-moderate and severe groups according to the initial EDSS score (≤3 or >3, respectively) to investigate predictive factors that may affect the severity of neurological deficits at the time of disease onset.

The severity of neurological deficits in patients with MOGAD was evaluated using the EDSS score, which ranges between 0 and 10, with higher scores indicating more severe disability. Scanning was performed using a 3.0 T MRI scanner. Blood samples were collected from patients during the early morning of the second day after admission. Antibodies against MOG, AQP4, and autoimmune encephalitis-related antibodies from blood or CSF samples were detected using CBA. We assumed that the MOG status was positive when antibody titers were higher than 1:10. Anti-MOG antibody titers varied from 1:10 to 1:1,000++. Antibody titers >1:1,000 were described as 1:1,000+ or 1:1,000++.

### 2.3. Statistical analyses

Continuous data conforming to normal distribution were expressed as mean ± standard deviation; otherwise, they were shown as median ± interquartile range. Categorical variables were presented as frequencies (percentages, %). When continuous data were normally distributed, a *t*-test for independent samples was used for comparisons between the groups. When data were not normally distributed, the Mann–Whitney *U*-test was used for comparison between the groups. Categorical data were compared using the chi-square test or Fisher's exact test. To investigate the relationship between FAR and disease severity, we performed binary logistic regression and correlation analysis: a univariate logistic regression analysis model was used to identify potential predictors for the severity of neurological deficits at disease onset, and multivariate logistic regression analysis was performed to determine the independent effect of FAR on the severity of neurological deficits at the onset of MOGAD; the basic model of multivariate logistic regression analysis included variables with a significance level of *P* < 0.1 in the univariate logistic regression analysis. The adjusted model included variables that are clinically considered to affect initial EDSS (including age at onset and sex) to analyze the stability of the correlation between the FAR level and severity of neurological deficits. The Spearman correlation analysis was used to detect the correlation between FAR and EDSS scores. The diagnostic value of FAR for the severity of neurological deficits was analyzed using receiver operating characteristic (ROC) curve analysis and the area under the curve (AUC). A *P*-value of < 0.05 was considered to be statistically significant. The Statistical Package for the Social Sciences 21.0 version software was used for statistical analysis.

## 3. Results

### 3.1. Demographic and clinical characteristics

As shown in Table 1, 82 patients (women, *n* = 38; men, *n* = 44) meeting the inclusion criteria were included. The mean age at disease onset was 20.00 (9.00, 33.00) years old, and the first MOGAD attack age was 1–64 years old. There were 36 patients in the pediatric group (<18 years old), and 15 (41.7%) were girls. The adult group (≥18 years old) comprised of 46 patients and 23 (50%) were women. The difference in sex between the two groups was not statistically significant (*P* > 0.05). The clinical manifestations of this disease were diverse. Comparison between the groups showed that the incidence of fever was higher in patients <18 years old than in patients ≥18 years old, and the difference was statistically significant (*P* < 0.05); paresthesia was more common in patients ≥18 years old than in patients <18 years old, and the difference was statistically significant (*P* < 0.05). The remaining clinical manifestations were not statistically different between the two groups (*P* > 0.05). Among the clinical phenotypes, ADEM was more common in the pediatric group (41.7%), and ON or TM was more frequent in the adult group (32.6%, 26.1%). The differences between the two groups were statistically significant (*P* < 0.05). The initial and discharge EDSS scores were not significantly different between the two groups (*P* > 0.05; Table 1).

**Table 1:** Clinical characteristics of patients in different age groups.

**Clinical characteristics**	**Participants (*n* = 82)**	**Pediatric group (n = 36)**	**Adult group (*n* = 46)**	***χ^2^/Z***	***P*-value**
Age	20.00 (9.00–33.00)	8.00 (5.25–14.00)	31.00 (25.00–41.00)	−7.740	**< 0.001**
Sex, female	38 (46.3%)	15 (41.7%)	23 (50.0%)	0.564	0.453
**Clinical manifestations**					
Paresthesia	19 (23.20%)	4 (11.1%)	15 (32.6%)	5.243	**0.022**
Paralysis	26 (31.7%)	9 (25.0%)	17 (37.0%)	1.333	0.248
Bladder/bowel dysfunction	14 (17.1%)	3 (8.3%)	11 (23.9%)	3.462	0.063
Blurred vision	31 (37.8%)	10 (27.8%)	21 (45.7%)	2.744	0.098
Eye pain	11 (13.4%)	6 (16.7%)	5 (10.9%)	0.192	0.661
Nausea/vomiting	10 (12.2%)	7 (19.4%)	3 (6.5%)	2.058	0.151
Fever	27 (32.9%)	18 (50.0%)	9 (19.6%)	8.470	**0.004**
Headache	27 (32.9%)	13 (36.1%)	14 (30.4%)	0.295	0.587
Dysarthria	2 (2.4%)	2 (5.6%)	0		0.190
Facial paralysis	7 (8.5%)	4 (11.1%)	3 (6.5%)	0.116	0.734
Disturbance of consciousness	6 (7.3%)	3 (8.3%)	3 (6.5%)	0.000	> 0.999
Seizures	17 (20.7%)	9 (25.0%)	8 (17.4%)	0.711	0.399
**Clinical phenotype**					
ON	19 (23.2%)	4 (11.1%)	15 (32.6%)	5.243	**0.022**
TM	14 (17.1%)	2 (5.6%)	12 (26.1%)	6.013	**0.014**
ON+TM	9 (11.0%)	4 (11.1%)	5 (10.9%)	0.000	> 0.999
Meningeal/brainstem encephalitis	19 (23.2%)	11 (30.6%)	8 (17.4%)	1.966	0.161
ADEM	21 (25.6%)	15 (41.7%)	6 (13.0%)	8.685	**0.003**
**EDSS**					
Initial EDSS	3.5 (2.0–4.625)	3.5 (2.0–4.5)	3.25 (2.5–5.0)	−0.522	0.601
Discharge EDSS	2.0 (0.0–3.0)	1.75 (0.0–2.88)	2.0 (0.0–3.125)	−0.801	0.423

Values are presented as mean ± standard deviation, number (percentage), or median (interquartile range).

ADEM, acute disseminated encephalomyelitis; ON, optic neuritis; TM, transverse myelitis; EDSS, expanded disability status scale.

Statistical significance with a two-sided *P*-value of < 0.05.

Bold it when *P*-value < 0.05.

### 3.2. Laboratory examinations

Neutrophil count, prothrombin time (PT), activated partial thromboplastin time (aPTT), and globulin levels were significantly higher in pediatric patients than in adult patients (*P* < 0.05). Thrombin time (TT) and urea were significantly higher in patients ≥18 years old than in those <18 years old (*P* < 0.05; Table 2). A total of eight (22.2%) patients <18 years old had positive CSF oligoclonal bands (OBs), compared with five (10.9%) patients ≥18 years old, with no significant between-group difference (*P* > 0.05). Anti-N-methyl-D-aspartate receptor (anti-NMDAR) antibodies were present in the first episode of MOGAD in two (5.6%) patients in the pediatric group and five patients (10.9%) in the adult group, with no statistically significant difference between the two groups (*P* > 0.05; Table 2).

**Table 2:** Laboratory data in different age groups.

**Variables**	**Participants (*n* = 82)**	**Pediatric group (*n* = 36)**	**Adult group (*n* = 46)**	**χ2/Z/*t***	***P*-value**
Neutrophil count (×10^9^/L)	5.46 (3.78–8.52)	6.57 (4.55–10.28)	4.91 (3.61–7.41)	−2.518	**0.012**
Lymphocyte count (×10^9^/L)	1.95 (1.25–2.52)	2.01 (1.29–2.85)	1.90 (1.22–2.42)	−0.762	0.446
Monocyte count (×10^9^/L)	0.47 (0.36–0.66)	0.45 (0.31–0.70)	0.47 (0.36–0.62)	−0.033	0.974
Eosinophil count (×10^9^/L)	0.09 (0.03–0.17)	0.09 (0.01–0.21)	0.08 (0.03–0.15)	−0.145	0.885
PT (s)	11.20 (10.60–11.59)	11.43 (10.95–12.00)	11.10 (10.50–11.40)	−3.036	**0.002**
aPTT (s)	29.20 (27.70–31.81)	29.91 (28.14–32.89)	28.50 (27.10–31.38)	−1.991	**0.047**
TT (s)	15.41 ± 1.88	14.84 ± 1.43	15.86 ± 2.08	−2.634	**0.010**
Fibrinogen (g/L)	3.16 ± 0.77	3.34 ± 0.77	3.01 ± 0.75	1.909	0.060
D-dimer (mg/L)	0.13 (0.07–0.21)	0.12 (0.07–0.23)	0.16 (0.07–0.19)	−0.070	0.944
TC (mmol/L)	3.80 (3.31–4.35)	3.82 (3.15–4.26)	3.78 (3.44–4.66)	−0.916	0.360
Triglycerides (mmol/L)	0.94 (0.62–1.19)	0.97 (0.55–1.13)	0.92 (0.66–1.33)	−0.388	0.698
HDL (mmol/L)	1.09 (0.96–1.26)	1.13 (0.99–1.25)	1.03 (0.94–1.29)	−1.201	0.230
LDL (mmol/L)	2.26 (1.90–2.68)	2.24 (1.86–2.61)	2.31 (1.93–2.93)	−1.290	0.197
Urea (mmol/L)	4.00 ± 1.21	3.52 ± 1.10	4.39 ± 1.18	−3.423	**0.001**
UA (μmol/L)	251.65 ± 84.39	235.72 ± 87.68	264.11 ± 80.49	−1.524	1.131
Albumin (g/L)	42.26 ± 4.21	42.43 ± 3.74	42.12 ± 4.59	0.330	0.742
Globulin (g/L)	25.82 ± 3.29	26.79 ± 3.24	25.05 ± 3.16	2.442	**0.017**
Positive oligoclonal band	13 (15.9%)	8 (22.2%)	5 (10.9%)	1.951	0.162
CSF Anti-NMDAR antibody	7 (8.5%)	2 (5.6%)	5 (10.9%)	0.208	0.648

Values are presented as mean ± standard deviation, number (percentage), or median (interquartile range).

CSF, cerebrospinal fluid; antiNMDAR, antiN-methyl-D-aspartate receptor; TC, total cholesterol; HDL, high-density lipoprotein cholesterol; LDL, low-density lipoprotein cholesterol; PT, Prothrombin time; TT, Thrombin time; aPTT, activated partial thromboplastin time; UA, uric acid.

Statistical significance with a two-sided *P*-value of < 0.05.

Bold it when *P*-value < 0.05.

### 3.3. Magnetic resonance imaging features

As shown in Table 3, 82 patients underwent cranial MRI in the acute stage, and patients <18 years old had significantly more lesions in the basal ganglia than those in patients ≥ 18 years old (*P* < 0.05). Spinal cord MRI was performed in 61 patients, among whom the cervical and thoracic segments of the spinal cord were more commonly involved, with no statistically significant difference in the distribution of lesions between the two groups (*P* > 0.05). As shown in Table 4, patients with EDSS > 3 had significantly more lesions in the brainstem and cerebellum than patients with EDSS ≤ 3 (*P* < 0.05).

**Table 3:** Localization of brain magnetic resonance imaging lesions in patients according to age groups.

**Lesion location**	**Participants (*n* = 82)**	**Pediatric group (*n* = 36)**	**Adult group (*n* = 46)**	***χ^2^***	***P*-value**
Optic nerve	13 (15.9%)	4 (11.1%)	9 (19.6%)	1.082	0.298
Cortical/subcortical	40 (48.8%)	21 (58.3%)	19 (41.3%)	2.344	0.126
Periventricular	18 (22.0%)	10 (27.8%)	8 (17.4%)	1.272	0.259
Corpus callosum	5 (6.1%)	1 (2.8%)	4 (8.7%)	0.418	0.518
Basal ganglia	10 (12.2%)	10 (27.8%)	0	12.074	**0.001**
Internal capsule	1 (1.2%)	0	1 (2.2%)		> 0.999
Thalamus	17 (20.7%)	10 (27.8%)	7 (15.2%)	1.939	0.164
Hippocampus	1 (1.2%)	1 (2.8%)	0		0.439
Brainstem	25 (30.5%)	13 (36.1%)	12 (26.1%)	0.958	0.328
Cerebellum	13 (15.9%)	7 (19.4%)	6 (13.0%)	0.620	0.431
Cervical spinal cord	26/61 (42.6%)	12/30 (40.0%)	14/31 (45.2%)	0.166	0.684
Thoracic spinal cord	24/61 (39.3%)	14/30 (46.7%)	10/31 (32.3%)	1.326	0.249
Lumbar spinal cord	3/61 (4.9%)	2/30 (6.7%)	1/31 (3.2%)	0.001	0.977

Values are presented as number (percentage).

Statistical significance with a two-sided *P*-value of < 0.05.

Bold it when *P*-value < 0.05.

**Table 4:** Localization of brain magnetic resonance imaging lesions in patients according to initial expanded disability status scale (EDSS) scores.

**Lesion location**	**Participants (*n* = 82)**	**EDSS ≤ 3 (*n* = 40)**	**EDSS>3 (*n* = 42)**	***χ^2^***	***P*-value**
Optic nerve	13 (15.9%)	5 (12.5%)	8 (19.0%)	0.658	0.417
Cortical/subcortical	40 (48.8%)	21 (52.5%)	19 (45.2%)	0.432	0.511
Periventricular	18 (22.0%)	11 (27.5%)	7 (16.7%)	1.403	0.236
Corpus callosum	5 (6.1%)	2 (2.4%)	3 (7.1%)	0	> 0.999
Basal ganglia	10 (12.2%)	4 (10.0%)	6 (14.3%)	0.065	0.799
Internal capsule	1 (1.2%)	0	1 (2.4%)		> 0.999
Thalamus	17 (20.7%)	6 (15.0%)	11 (26.2%)	1.561	0.211
Hippocampus	1 (1.2%)	1 (2.5%)	0		0.488
Brainstem	25 (30.5%)	8 (20.0%)	17 (40.5%)	4.053	**0.044**
Cerebellum	13 (15.9%)	3 (7.5%)	10 (23.8%)	4.085	**0.043**
Cervical spinal cord	26/61 (42.6%)	9/27 (33.3%)	17/34 (50.0%)	1.709	0.191
Thoracic spinal cord	24/61 (39.3%)	9/27 (33.3%)	15/34 (44.1%)	0.733	0.392
Lumbar spinal cord	3/61 (4.9%)	2/27 (7.4%)	1/34 (2.9%)	0.042	0.837

Values are presented as numbers (percentages).

Statistical significance with a two-sided *P*-value of < 0.05.

Bold it when *P*-value < 0.05.

### 3.4. The relationship between FAR and disease severity

As shown in Table 5, there was no significant difference between the two groups regarding sex, age at onset, neutrophil count, lymphocyte, monocyte, eosinophil, PT, aPTT, TT, D-dimer, total cholesterol (TC), triglyceride, low-density lipoprotein (LDL), urea, globulin, positive OBs, and positive anti-NMDAR antibodies (*P* > 0.05). The levels of ALB, uric acid (UA), and high-density lipoprotein (HDL) were significantly higher in patients with EDSS ≤ 3 than in those with EDSS >3, while the levels of FIB and FAR were significantly lower in patients with EDSS ≤ 3 than in those with EDSS >3 (*P* < 0.05).

**Table 5:** Laboratory data according to the initial expanded disability status scale (EDSS) score.

**Variables**	**EDSS ≤ 3 (*n* = 40)**	**EDSS >3 (*n* = 42)**	***χ2/Z/t***	***P*-value**
Sex, female	16 (40.0)	22(52.4)	1.263	0.261
Age, years	20.00 (11.50–33.00)	21.00 (6.00–33.25)	−0.719	0.472
Neutrophil count (×10^9^/L)	5.32 (3.70–7.93)	5.79 (4.12–9.25)	−0.974	0.330
Lymphocyte count (×10^9^/L)	1.99 (1.52–2.43)	1.87 (1.14–2.61)	−0.923	0.356
Monocyte count (×10^9^/L)	0.46 (0.37–0.65)	0.47 (0.31–0.67)	−0.548	0.584
Eosinophil count (×10^9^/L)	0.10 (0.04–0.18)	0.05 (0.01–0.18)	−1.255	0.209
PT (s)	11.25 (10.70–11.60)	11.16 (10.60–11.52)	−0.576	0.565
aPTT (s)	28.85 (27.83–31.58)	29.50 (27.28–32.00)	−0.747	0.455
TT (s)	15.51 ± 1.87	15.32 ± 1.92	0.470	0.640
Fibrinogen (g/L)	2.88 ± 0.66	3.42 ± 0.78	−3.338	**0.001**
D-dimer (mg/L)	0.10 (0.07–0.19)	0.17 (0.08–0.23)	−1.562	0.118
TC (mmol/L)	3.66 (3.18–4.22)	3.97 (3.43–4.44)	−1.132	0.258
Triglycerides (mmol/L)	0.92 (0.57–1.08)	0.96 (0.67–1.23)	−0.719	0.472
HDL (mmol/L)	1.12 (0.98–1.35)	1.03 (0.88–1.18)	−2.00	**0.046**
LDL (mmol/L)	2.10 (1.87–2.53)	2.49 (1.94–2.87)	−1.619	0.105
Urea (mmol/L)	4.21 ± 1.20	3.81 ± 1.21	1.522	0.132
UA (μmol/L)	271.43 ± 92.93	232.81 ± 71.49	2.115	**0.038**
Albumin (g/L)	43.40 ± 3.36	41.17 ± 4.68	2.467	**0.016**
Globulin (g/L)	26.02 ± 3.42	25.63 ± 3.20	0.529	0.598
FAR (%)	6.46 (5.66–7.74)	7.78 (6.93–9.97)	−3.887	**< 0.001**
Positive oligoclonal band	8 (20.0)	5 (11.9)	1.006	0.316
CSF Anti-NMDAR antibody	5 (12.5)	2 (4.8)	0.736	0.391

Data are presented as mean ± standard deviation, number (percentage), or median (interquartile range).

EDSS, Expanded Disability Status Scale; CSF, cerebrospinal fluid; anti-NMDAR, anti-N-methyl-D-aspartate receptor; TC, total cholesterol; HDL, high-density lipoprotein cholesterol; LDL, low-density lipoprotein cholesterol; PT, prothrombin time; TT, thrombin time; aPTT, activated partial thromboplastin time; UA, uric acid; FAR, fibrinogen-to-albumin ratio.

Statistical significance with a two-sided *P*-value of < 0.05.

Bold it when *P*-value < 0.05.

As shown in Table 6, univariate logistic regression analysis showed that FIB (odds ratio (OR) = 2.900; 95% confidence interval CI = 1.438–5.849; *P* = 0.003), ALB (OR = 0.871; 95% CI = 0.774–0.979; *P* = 0.020), uric acid (OR = 0.994; 95% CI = 0.989–1.000; *P* = 0.042), HDL levels (OR = 0.171; 95% CI = 0.029–0.993; *P* = 0.049), and FAR (OR = 1.827; 95% CI = 1.302–2.564; *P* < 0.001) were significantly associated with the initial EDSS score.

**Table 6:** Univariate logistic regression for assessing the association of fibrinogen-to-albumin ratio with disease severity.

**Factors**	**Univariate logistic regression OR (95%CI)**	***P*-value**
Age, years	0.993 (0.966–1.021)	0.639
Sex, female	0.606 (0.252–1.455)	0.262
Neutrophils	1.080 (0.955–1.222)	0.222
Lymphocytes	0.801 (0.488–1.314)	0.379
Monocytes	0.685 (0.120–3.912)	0.670
Eosinophils	1.219 (0.082–18.124)	0.885
PT	0.962 (0.862–1.073)	0.487
aPTT	1.068 (0.959–1.189)	0.231
TT	0.945 (0.750–1.192)	0.635
Fibrinogen	2.900 (1.438–5.849)	**0.003**
D-dimer	8.196 (0.298–225.488)	0.214
TC	1.139 (0.699–1.855)	0.602
Triglycerides	1.490 (0.648–3.428)	0.348
HDL	0.171 (0.029–0.993)	**0.049**
LDL	1.200 (0.669–2.151)	0.541
Urea	0.751 (0.516–1.093)	0.135
UA	0.994 (0.989–1.000)	**0.042**
Albumin	0.871 (0.774–0.979)	**0.020**
Globulin	0.964 (0.844–1.102)	0.594
FAR	1.827 (1.302–2.564)	**< 0.001**
Positive oligoclonal band	0.541 (0.161–1.819)	0.320
CSF Anti-NMDAR antibody	0.350 (0.064–1.919)	0.227

FAR, fibrinogento-albumin ratio; CSF, cerebrospinal fluid; EDSS, expanded disability status scale scores; MOGAD, MOG-IgG associated disorders; OR, odds ratio; CI, confidence interval; TC, total cholesterol; HDL, high-density lipoprotein cholesterol; LDL, low-density lipoprotein cholesterol; PT, prothrombin time; TT, thrombin time; aPTT, activated partial thromboplastin time; UA, uric acid.

Statistical significance with a two-sided *P*-value of < 0.05.

Bold it when *P*-value < 0.05.

As shown in Table 7, FAR (OR = 1.693; 95% CI = 1.186–2.417; *P* = 0.004) was correlated with the initial EDSS score in the basic model corrected for other risk factors. In the adjusted model corrected for related factors (age at admission and sex), FAR was still independently associated with the severity of neurological deficits at the onset of MOGAD (OR = 1.717; 95% CI = 1.191–2.477; *P* = 0.004).

**Table 7:** Multivariate logistic regression for assessing the association of fibrinogen-to-albumin ratio with disease severity.

**Factors**	**Multivariate logistic regression**			
	**Basic model** ^a^		**Adjusted model** ^b^	
	**OR (95%CI)**	***P*** **-value**	**OR (95%CI)**	***P*** **-value**
Sex, female			0.552 (0.183–1.667)	0.292
Age at onset			1.000 (0.967–1.033)	0.987
FAR	1.693 (1.186–2.417)	**0.004**	1.717 (1.191–2.477)	**0.004**
UA	0.995 (0.989–1.002)	0.149	0.997 (0.990–1.003)	0.329
HDL	0.266 (0.037–1.897)	0.187	0.233 (0.030–1.802)	0.163

FAR, fibrinogen-to-albumin ratio; UA, uric acid; HDL, high-density lipoprotein cholesterol; EDSS, expanded disability status scale; OR, odds ratio; CI, confidence interval.

a Basic model: Variables with a *P*-value of < 0.1 in the univariate logistic regression analysis were included in the multivariate model.

a Adjusted model: Variables with a *P*-value of < 0.1 in the univariate logistic regression analysis or variables clinically assumed to have an impact on initial EDSS (including age at onset and sex) were included in the adjusted model.

Statistical significance with a two-sided *P*-value of < 0.05.

Bold it when *P*-value < 0.05.

**Figure 1:**
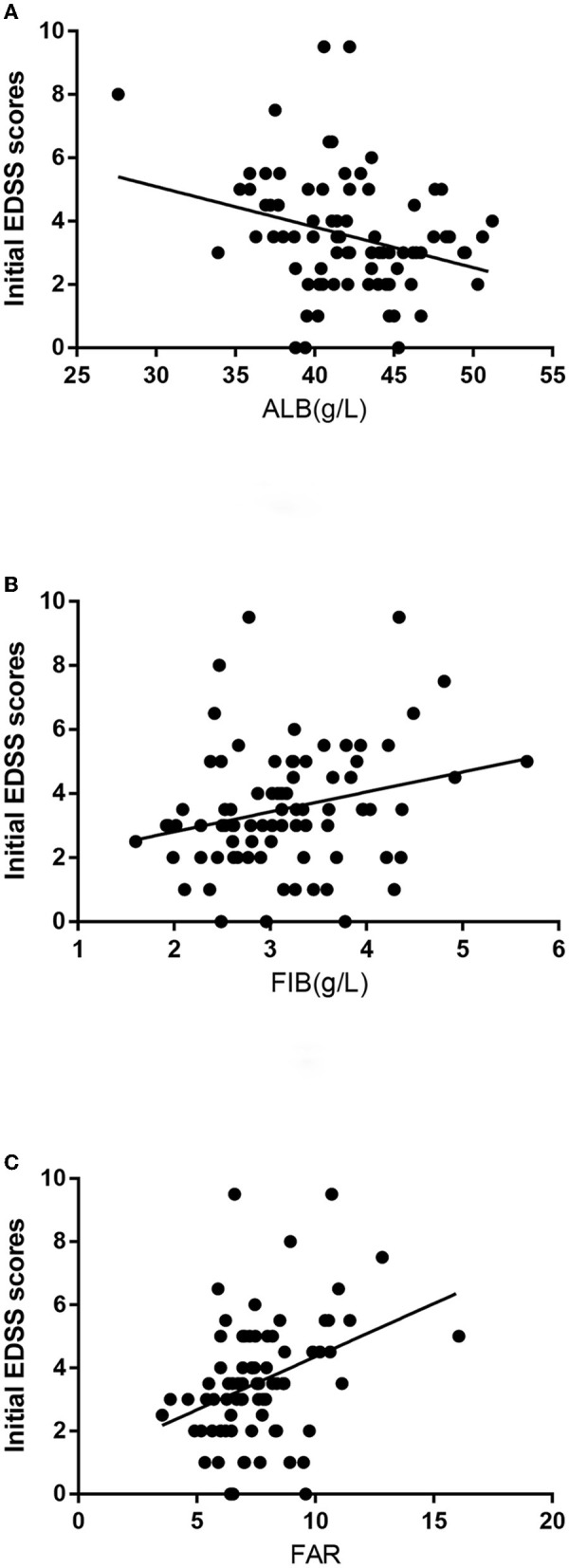
**(A)** The correlation between albumin (ALB) levels and the initial expanded disability status scale (EDSS) scores is shown. **(B)** The correlation between fibrinogen (FIB) levels and the initial EDSS scores is shown. **(C)** The correlation between FIB-to-ALB ratio (FAR) levels and the initial EDSS scores is shown.

The Spearman correlation analysis revealed that FAR (*r* = 0.359, *P* = 0.001) and FIB (*r* = 0.257, *P* = 0.020) were positively correlated with the initial EDSS score, whereas ALB levels (*r* = −0.258, *P* = 0.019) were negatively correlated with the initial EDSS score (Figure 1).

**Figure 2:**
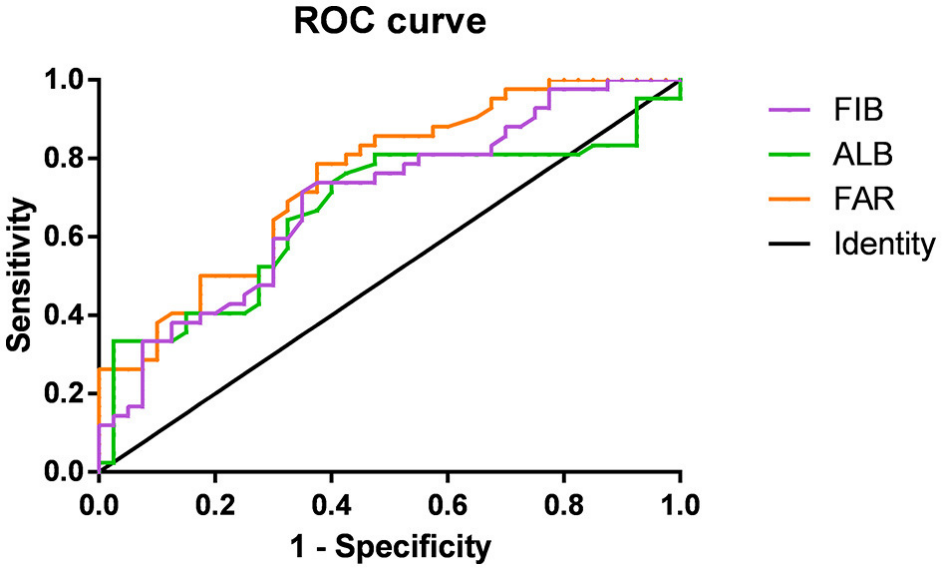
Receiver-operating characteristic (ROC) curves showing the association between fibrinogen (FIB; purple), albumin (ALB; green), FIB-to-ALB ratio (FAR; orange), and the severity of neurological dysfunction at the onset of MOG-IgG–Associated Disorders (MOGAD).

Figure 2 shows the association of FIB, ALB, and FAR with MOGAD severity at onset based on ROC curve analysis. When the ALB cut-off value was 43.15 g/L, the AUC was 0.665 (95% CI = 0.545–0.786, *P* = 0.010), with a sensitivity of 73.8% and specificity of 60%. At a FIB cutoff value of 3.035 g/L, the AUC was 0.694 (95% CI = 0.580–0.807, *P* = 0.003), with a sensitivity of 71.4% and specificity of 65%. The AUC was 0.749 (95% CI = 0.645–0.853, *P* < 0.001), with a sensitivity of 78.6% and specificity of 62.5% when the FAR cutoff value was 6.92%. The AUC of FAR was significantly higher than that of FIB and ALB.

## 4. Discussion

In this study, a retrospective analysis was conducted to compare clinical, laboratory, and MRI findings between pediatric and adult patients with first-episode MOGAD and explore the risk factors that may predict severity at disease onset. Our study demonstrated that clinical features were different between patients of different ages. The most common disease spectrum in patients <18 years old was ADEM, whereas ON or TM was commonly found in patients ≥18 years old. Cortical/paracortical lesions were the most common lesions in both pediatric and adult patients. The FAR level at admission was significantly associated with the severity of neurological deficits. FAR has a good clinical value for assessing the severity of first-episode MOGAD.

The sex difference between MOGAD patients in this study was not statistically significant, and the male-to-female ratio was 1.16:1, which is consistent with previous studies, and these studies have shown a male-to-female ratio of approximately 1:1 (18), which differs from the female bias often observed in other immune-mediated/autoimmune diseases, including AQP4-IgG + NMOSD (1:9.2) and MS (1:3) (19–21). In the present study, the most common clinical phenotype at onset in patients ≥18 years old was ON, and ADEM was the most common phenotype in the pediatric group, which was consistent with previous studies (20, 22, 23). Cortical/paracortical lesions on encephalic MRI in patients were the most common lesions observed, while spinal cord injuries in this study mainly involved the cervical or thoracic segments, consistent with previous studies (7, 25). In the current study, the incidence of OBs was 15.9%, which was consistent with previous studies (<20%) (7, 20).

Autoimmune encephalitis (AE) causes severe neurologic symptoms in patients of all ages, and advances in AE diagnosis have led to the identification of more antibodies causing brain inflammation, so antibodies to MOG and NMDAR have been reported with increasing frequency in AE (24). In this study, the incidence of anti-NMDAR antibody positivity was 8.5%, which is similar to that reported in previous studies (25), and none of them met the diagnostic criteria for anti-NMDAR AE.

In this study, correlation analysis showed that FIB, ALB, and FAR were all correlated with the initial EDSS score, and binary logistic regression analysis showed that FAR was an independent risk factor for the severity of MOGAD. ROC curve analysis showed that FAR was a good indicator for assessing the severity of disease at admission.

Fibrinogen can enter the CNS after the destruction of the blood–brain barrier (BBB) and is related to neuroinflammation, neuronal injury, and immune cell recruitment in the nervous system. Fibrin is a crucial factor in inflammatory demyelination, neurodegeneration, and inhibition of CNS repair (26). At sites of BBB disruption, FIB is converted to inflammatory fibrin, which can induce the release of reactive oxygen species (ROS) and M1-like activation of microglia and macrophages (14, 27). In animal models of MS, FIB enters the CNS parenchyma *via* the damaged BBB, which is deposited in the form of insoluble fibrin in the brain tissue (11) and binds to the CD11b/CD18 integrin receptor, which induces ROS release in the microglia and recruitment of peripheral macrophages and T cells, leading to autoimmune demyelination and axonal destruction (14, 26–28). FIB is a potent exogenous inhibitor of oligodendrocyte progenitor cell differentiation and remyelination (29). Destruction of BBB in the acute phase of MOGAD suggests that FIB may contribute to the acute inflammatory response through BBB. The level of FIB is positively correlated with the severity of the inflammatory response and disease. A correlation was found between FIB levels and the severity of coronary lesions in patients with the acute coronary syndrome (30). In addition, FIB levels in patients with NMOSD were correlated with disease severity (31).

Albumin is associated with several critical physiological functions, such as the maintenance of plasma colloid oncotic pressure, regulation of microvascular permeability, metabolism, lipid transport, antioxidant, anti-inflammatory effect, and immunomodulatory effect (16, 32, 33). ALB is thought to be associated with many neurological disorders because of its ability to modulate the hemodynamic properties of the cerebral circulation and its direct neuroprotective functions in the neuronal and glial cells. In experimental ischemic stroke models, exogenous human serum ALB is neuroprotective by promoting amelioration of brain swelling, preventing postischemic thrombosis, antioxidation, hemodilution, and improving microvascular hemoperfusion to increase the perfusion volume of the ischemic tissue. In experimental models of Alzheimer's disease, it is thought to be neuroprotective by inhibiting aggregation and enhancing the clearance of β-amyloid (16). ALB is a valuable biomarker for several diseases. Studies have found that a lower ALB level was associated with an increased risk of mortality in cardiovascular disease and carotid atherosclerosis (34, 35). Low ALB levels were also observed in some autoimmune diseases of the nervous system, such as Guillain–Barre syndrome, myasthenia gravis, and NMOSD (36–38).

Fibrinogen and ALB are vital factors in the coagulation system and reliable indicators for nutritional status and inflammation. FAR, which incorporates these two indicators, may have a better predictive value than any single biomarker. As a novel inflammatory serological marker, FAR has promising predictive ability in a variety of diseases, such as various tumors (pancreatic, esophageal, and liver cancers) (39–41), cardiovascular diseases (30, 42), and in estimating the risk of hemorrhagic transformation in patients with acute ischemic stroke (43). However, a study including 40 patients with MS found no statistically significant correlation between the mean EDSS score and peripheral blood FAR (*P* > 0.05) (44). The relationship between peripheral blood FAR and inflammatory demyelinating diseases of the CNS requires further exploration.

This study had several limitations. First, as a retrospective study, the number of patients included was relatively small. Data were acquired from a single center and region, which may inevitably lead to subjective selection bias. Second, our study only included results from the early stage of admission, and long-term prognostic information was limited. Long-term follow-up data must be refined. Third, this study recorded the FAR level only once at the time of admission, and studies examining the dynamic changes of FAR over time are limited.

In summary, the present study showed that the clinical and imaging manifestations of MOGAD differed between patients of different ages. Patients with EDSS ≤ 3 had significantly lower FAR levels than those with EDSS >3, and FAR was positively correlated with the initial EDSS score. In addition, the potential role of FIB and ALB in disease progression may be used clinically to provide new treatment approaches against the disease. Detection of FAR is simple, noninvasive, and reproducible and is expected to become a relatively novel and reliable laboratory indicator for assessing the severity at disease onset in patients with first-episode MOGAD.

## Data availability statement

The raw data supporting the conclusions of this article will be made available by the authors, without undue reservation.

## Ethics statement

The studies involving human participants were reviewed and approved by the Ethics Committee of First Affiliated Hospital of Zhengzhou University. Written informed consent for participation was not required for this study in accordance with the national legislation and the institutional requirements. Written informed consent was not obtained from the individual(s), nor the minor(s)' legal guardian/next of kin, for the publication of any potentially identifiable images or data included in this article.

## Author contributions

YL contributed to the project conception, performed the statistical analysis, and wrote the manuscript. SW interpreted the data and prepared the figures. PL and JM screened and extracted the data. XL and JY supervised this study. All authors have made intellectual contributions to the manuscript and have approved of its submission.
